# Mediterranean Diet Adherence and Nutritional Status in Dalmatian Diabetic Hypertensive Patients Regarding Presence of Chronic Kidney Disease—Is There Any Difference?

**DOI:** 10.3390/ijerph19042293

**Published:** 2022-02-17

**Authors:** Dora Bučan Nenadić, Josipa Radić, Ela Kolak, Marijana Vučković, Ivana Novak, Marija Selak, Mislav Radić

**Affiliations:** 1 Department of Nutrition and Dietetics, University Hospital Centre Split, 21000 Split, Croatia; dorabucan@gmail.com (D.B.N.); elakolak93@gmail.com (E.K.); marija.selak3@gmail.com (M.S.); 2 School of Medicine, University of Split, 21000 Split, Croatia; mislavradic@gmail.com; 3 Division of Nephrology and Dialysis, Department of Internal Medicine, University Hospital Centre Split, 21000 Split, Croatia; mavuckovic@kbsplit.hr (M.V.); ivana.i.novak@gmail.com (I.N.); 4 Division of Clinical Immunology and Rheumatology, Department of Internal Medicine, University Hospital of Split, 21000 Split, Croatia

**Keywords:** dietary habits, Mediterranean diet, diabetes, hypertension, chronic kidney disease, body composition, Dalmatian

## Abstract

In recent years, the Mediterranean diet has emerged as one of the dietary patterns that could have positive effects on overall health as well in the treatment of non-communicable chronic diseases. The aim of this cross-sectional study was to determine differences in adherence to the Mediterranean diet (MeDi) and nutritional status in patients with type 2 diabetes mellitus (T2DM) and arterial hypertension (AH) regarding the presence of chronic kidney disease (CKD). Two hundred and forty-eight Dalmatian diabetic hypertensive patients (DDHP) were included, and 164 (66.1%) of them had CKD. Data about anthropometric parameters, clinical and laboratory parameters, as well as lifestyle questionnaire and Mediterranean Diet Serving Score (MDSS) were collected for each study participant. Furthermore, body composition was assessed using MC-780 Multi Frequency Segmental Body Mass Analyzer (Tanita). Body mass index (BMI) as well as waist-to-hip ratio (WHR) and waist-to-height ratio (WHtR) were calculated. Results showed that only 8.9% of DDHP were adherent to the MeDi without significant differences regarding the presence of CKD. Therefore, only 9.1% of participants with CKD were adherent to the MeDi. Dietary recommendations were received by 52.8% of DDHP and 49.4% with CKD, while only 12.8% of those with CKD were adherent to the given recommendations. The results showed that 88.3% of DDHP and 87.8% of the DDHP with CKD were overweight or obese. Statically significant lower frequency of nut intake suggested by the MeDi was found in those participants with CKD (*p* = 0.02). Therefore, the significant associations between adherence to each MeDi component as well as MDSS score with the development of CKD among all study subjects were not found. In conclusion, the results showed a low level of nutritional care in our region and low adherence to MeDi among DDHP. According to the results, there is an urgent need to improve nutritional care in our region, with a special focus on the MeDi for this especially vulnerable population of patients.

## 1. Introduction

Arterial hypertension (AH) is the leading cause of death [1], and diabetes mellitus (DM) is among the top 10 causes of death around the world [2]. According to the latest CroDiab data, diabetes was the third leading cause of death, with a share of 7.8% in 2019 in Croatia [3]. In 2020, 310,212 people were diagnosed with diabetes, which makes up only 60% of all, so it is considered that the total number of people suffering from DM in Croatia has exceeded 500,000 [4]. Globally, DM and AH or their combination are the two most common causes of chronic kidney disease (CKD) [5] and cause about 80% of end-stage renal disease (ESRD) [2].

Although medicine gives various modalities of treatments that are effective, around 50% of these patients still do not have adequately regulated blood pressure or glycemic control [6]. Changes in lifestyle behaviors in recent years have corresponded with increases in many chronic non-communicable diseases. Healthy lifestyle behaviors, which include healthy diet and physical activity, are among the most promising and cost-effective strategies for reducing complications and premature death among people living with type 2 diabetes mellitus (T2DM) and AH [7]. The efficacy of the Mediterranean diet (MeDi) in the management as well as prevention of T2DM and AH is one of the most widely investigated dietary patterns in this area. 

In general, MeDi is considered as a traditional food-pattern characteristic for the geographical area surrounding the Mediterranean Sea, with certain differences regarding food choices and food preparation specific for each culture or country [8]. Moreover, it is recognized by the United Nations Educational, Scientific and Cultural Organization (UNESCO) as an “Intangible Cultural Heritage of Humanity” since it is an integral part of the heritage and culture as well as one of the healthiest dietary patterns due to the variety of plant-based foods and high intake of olive oil [9]. MeDi is characterized by high consumption of vegetables, fruits, grains, nuts, and legumes; moderate usage of fish, seafood, and dairy; as well as limited intake of meat and alcohol [10,11].

As such, it provides high-quality, nutrient-dense carbohydrates from whole grains, nuts, fruits, and vegetables as well as a low glycemic index, which has beneficial effects on glucose control, insulin responses, and blood lipids [10]. 

Nutritional interventions can slow the progression of the CKD in the earliest stages [12] but also delay the need for renal replacement therapy in later stages by carefully managing protein, potassium, phosphorus, sodium, and calcium intake [13]. Recommended intake of proteins according to the principles of the MeDi, which includes legumes, fish, and white meat, is in line with recommendations on protein intake for diabetic patients with CKD [14]. Moreover, reduced intake of red and processed meat contributes to a reduction of dietary sodium and phosphorus, which may have a positive effect on CKD progression [15]. Furthermore, the MeDi is rich in dietary fiber and as such provides between 30–50 g of fiber per day [10]. Increased dietary fiber intake lowers blood pressure and improves postprandial glycaemia and insulin sensitivity, and it is inversely associated with inflammation and mortality in diabetic patients with CKD [10]. 

According to the recent data, participants with the highest adherence to the MeDi had a 13–23% lower risk of developing diabetes in comparison to the participants with lower adherence to the MeDi [16]. Adherence to the MeDi, especially for those suffering from T2DM and CKD, has proven to be associated with reduction of cardiovascular risk, lower mortality risk, lower risk of CKD progression and lower levels of oxidative stress [10]. According to Gomes-Neto et al., greater adherence to the MeDi is associated with better kidney function outcomes in kidney transplant recipients (KTRs) [17], but low compliance to the MeDi principles was found in Dalmatian KTRs as demonstrated in our recent research [18]. 

To our knowledge, there is no study about MeDi adherence in patients with T2DM and AH in this specific region. Moreover, MeDi should be a traditional dietary pattern in Dalmatia, an actual Mediterranean region, and as such provide an abundance of local and seasonal food that may positively impact patient’s compliance to the given recommendations. Considering the overall beneficial impact of MeDi on the outcomes of chronic noncommunicable diseases, the aim of this cross-sectional study was to determine differences in adherence to the MeDi and nutritional status in patients with T2DM and AH from the Dalmatia region, Croatia, regarding the presence of CKD. 

## 2. Materials and Methods

### 2.1. Study Design and Population

**Figure 1:**
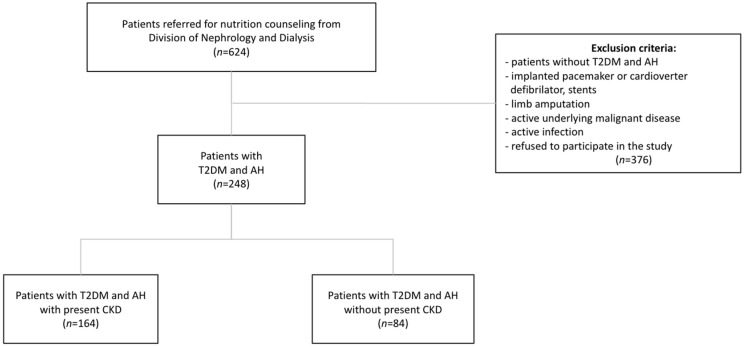
Study design. Abbreviations: CKD, chronic kidney disease; T2DM, type 2 diabetes mellitus; AH, arterial hypertension.

This research, designed as a cross-sectional study, was carried out at the Outpatient Clinic for Clinical Nutrition, Division of Nephrology and Dialysis, Department of Internal Medicine (Dept of Internal Medicine), University Hospital Centre Split, Croatia, between March 2019 and April 2020. Two hundred and forty-eight patients with a mean age of 68 (IQR 60–74) years and diagnosed with T2DM and AH were recruited during their regular visit to the nephrologist and dietitian. Therefore, 164 (66.1%) of DDHP had CKD as shown in Figure 1. Excluded from the study were those patients who met one of the following criteria: had implanted pacemaker or cardioverter defibrillator, stents, or limb amputation; had an active underlying malignant disease or active infection; and those who refused to participate in the study. All participants were informed about the purpose and nature of the study and gave written consent. 

The study protocol was accepted by the Ethics Committee of the University Hospital of Split on 29 March 2019 (Ur.no. 2181-147-01/06/M.S.-19-2, Class: 500-03/19-01/20.), and the study was conducted following the guidelines of the latest version of the declaration of Helsinki.

### 2.2. Body Composition and Anthropometric and Blood Pressure Measurement

For each study participant, body composition was assessed using MC-780 Multi Frequency Segmental Body Mass Analyzer (Tanita, Tokio, Japan). The scale sends an imperceptible current through the body and uses eight electrodes to measure the resistance of different tissues. This technology is called bioelectrical impedance analysis (BIA) and is used to estimate body mass (kg), muscle mass (kg), fat free mass (kg), fat mass (kg) and fat mass percentage (%), visceral fat, trunk fat mass (kg) and trunk fat mass percentage (%), skeletal muscle mass (kg) and skeletal muscle mass percentage (%), skeletal muscle index (SMI), and phase angle (°). The instructions for patients from the device manual were followed: not to take any food or liquid at least 3 h before the measurement, to urinate just before the measurement, and not to consume alcohol, eat or drink excessively, or exercise in an excessive way at least one day before the body composition measurement [19]. 

Non-stretchable, flexible body measuring tape was used to measure the circumference of the mid-upper arm (MUAC), hip (HC), and waist circumference (WC). The circumference of the upper arm is defined as relaxed, with the body stretched by the hand, with a measuring tape placed horizontally 1 cm above the middle of the upper arm. The waist circumference is defined above the navel in the standing position facing forward of the examinee, with the measuring tape set horizontally. Hip circumference is defined around the widest portion of the buttocks, with the tape parallel to the floor. Height was measured using a stadiometer [20]. For each study participant, body mass index (BMI) as well as waist-to-hip ratio (WHR) and waist-to-height ratio (WHtR) were calculated. 

Peripheral blood pressure measurements were performed using a digital sphygmomanometer Omron M6 Comfort HEM-7360-E Blood Pressure Monitor (Omron, Kyoto, Japan). The right-sized cuff was selected according to the upper arm circumference and positioned accurately. All participants were in a relaxing environment, comfortably seated with back and arm supported, feet flat on the ground, legs not crossed, and with an empty bladder. Blood pressure was measured for three times at one-minute intervals, and the average of the last two measurements was calculated. Data about peripheral systolic and diastolic blood pressure were obtained.

### 2.3. Lifestyle Questionnaire and Mediterranean Diet Serving Score

The lifestyle questionnaire consisting of a series of questions on sociodemographic information, dietary and smoking habits, as well as medication was administered by a qualified dietitian. Adherence to the MeDi pattern was evaluated by a semiquantitative food frequency questionnaire called the Mediterranean Diet Serving Score (MDSS), according to the recommended consumption frequency of fourteen (14) different food items as well as food groups (MeDi components). According to Monteagudo et al., MDSS is considered as a validated, easily applicable, and accurate tool for estimating adherence to the MeDi [21]. Based on the new Mediterranean food pyramid, three points were assigned for the recommended intake of food if consumed with every meal (cereals, olive oil, vegetables, and fruit). Next, two points were scored for the daily consumption of dairy products and nuts, and finally, one point was assigned for the recommended weekly intake of potatoes (≤3), legumes (≥2), eggs (2–4), poultry (2), red meat (<2), fish (≥2), sweets (≤2), and fermented beverages (1 and 2 glasses a day for females and males, respectively) [22]. 

Intake higher or lower than the recommendations for any MeDi components is given a total value of zero (0). According to the original study, the MDSS ranges from zero (0) to twenty-four (24), with optimal cut-off point set at ≥13.5 to determine the adherence to the MeDi [21], which was rounded up to 14 points because a single score of the test cannot be decimal number but a whole number only.

### 2.4. Medical History and Clinical and Laboratory Parameters

Data on the length of treatment forT2DM and AH as well as the other coexisting diseases, such as CKD, were obtained for each participant from their medical records. CKD was defined as estimated glomerular filtration rate (eGFR) < 60 mL/min/1.73 m^2^ or albuminuria > 300 mg/g.

All study participants underwent usual peripheral blood sampling, and a 24-h urine sample was taken on the same day of the body composition measuring. The collected data included following laboratory parameters: urea (mmol/L), creatinine (mmol/L), uric acid (mmol/L), serum albumin (g/L), hemoglobin (g/L), mean cellular volume (MCV) (fL), potassium (mmol/L), phosphates (mmol/L), calcium (mmol/L), glucose (mmol/L), hemoglobin A1c (HbA1c) (%), triglycerides (mmol/L), total cholesterol (mmol/L), low-density lipoprotein cholesterol (LDL) (mmol/L), eGFR using CKD-EPI (mL/min/1.73 m^2^), albuminuria (mg/g), proteinuria (mg/g), and albumin-to-creatinine ratio (ACR) (mg/g). 

Blood samples for analysis of serum levels of complement components were collected in standard test tubes without additives in our Laboratory of Medical Diagnostics and Biochemistry at the University Hospital of Split, Croatia, and 30-min later were centrifuged for 10 min at 1690× *g* on HERMLE Z400 centrifuge model (Hermle Labortechnik GmbH, Wehingen, Germany). For creatinine measurement, Jaffe method was used. A complete blood count was obtained using a hematology analyzer (Advia 120, Siemens, Erlangen, Germany). 

### 2.5. Statistical Analysis

Statistical analyses were performed using the statistical software the MedCalc Statistical Software version 18.2.1 (MedCalc Software bvba, Ostend, Belgium; http://www.medcalc.org (accessed on 12 April 2020) and SPSS (Statistics for Windows, Version 21.0, Armonk, NY, USA, IBM Corp. Released 2013). The categorical data are represented by absolute and relative frequencies. The variance of the category variables was tested by the chi-square test. The normality of the distribution of numeric variables was tested by the Shapiro–Wilk test. Numerical data were described by the median and the limits of the interquartile range. The differences between numeric variables were tested in case of deviation from the normal distribution by Mann–Whitney. Finally, bivariate regression analysis was performed to analyze the association between measured parameters, particularly the adherence to each MeDi component and MDSS with the development of kidney function. Results of logistic regression were provided as odds ratios (OR) with a 95% confidence interval (95% CI). Significance level was set at *p*-value < 0.05. 

## 3. Results

The recruited sample included a total of 248 DDHP, and 164 (66.1%) of them had CKD. Data about body composition and anthropometric and clinical parameters of all study participants (*n* = 248), including differences regarding the presence of CKD, are shown in Table 1.

Those DDHP with CKD were significantly older (*p* < 0.001) and had lower levels of diastolic blood pressure (*p* = 0.027) and lower BMI (*p* < 0.001). Additionally, a significantly higher proportion of DDHP with CKD were male (*p* = 0.004). Among participants with CKD, 42.1% were overweight, and 47.5% were obese, whereas the prevalence of overweight was 22.6%, and the prevalence of obesity was 66.7% among DDHP without CKD. Furthermore, given the anthropometric measures, statistically significant higher HC (*p* = 0.012) and WHR (*p* = 0.029) were observed in DDHP with CKD, while higher MUAC (*p* = 0.001), WC (*p* = 0.024), and WHtR (*p* = 0.047) were noticed in participants without CKD. Regarding body composition, non-CKD participants had significantly higher body fat (% and kg; *p* < 0.001) and trunk fat mass (% and kg; *p* = 0.001), whereas the CKD participants had significantly higher muscle mass (%; *p* < 0.001) and skeletal muscle mass (%; *p* < 0.001). 

Data about biochemical parameters of all study participants (*n* = 248), including differences regarding the presence of CKD, are shown in Table 2. Statistically significant differences were determined for the following parameters: erythrocyte count (*p* < 0.001), hemoglobin (Hb; *p* < 0.001), MCV (*p* = 0.004), urea (*p* < 0.001), creatinine (*p* < 0.001), eGFR (*p* < 0.001), potassium *(p* = 0.014), phosphorus *(p* = 0.007), albumin-to-creatinine ratio (ACR; *p* < 0.001), proteinuria (*p* < 0.001), albuminuria (*p* < 0.001), and uric acid *(p* = 0.006).

The dietary habits of the total study population according to the lifestyle questionnaire as well as differences among them regarding the presence of CKD are shown in Table 3. Out of all, 52.8% of DDHP had received dietary recommendations in the past, while just 14.9% adhered to those recommendations. DDHP with CKD had lower compliance to the given dietary recommendations in comparison with DDHP without CKD, but this difference did not reach a significant level. Moreover, 49.4% of DDHP with CKD received dietary recommendations in the past, but just 12.8% of them followed the given recommendations. Most of the study population (79.4%) had two to four meals per day, 8.1% of participants had one or two meals, and only 12.5% of them had more than four meals per day. Results showed that for 53% of all study subjects, family members are preparing meals. Therefore, in DDHP with CKD, significantly more often, family members are preparing meals when compared with those DDHP without CKD (*p* < 0.001). 

**Figure 2:**
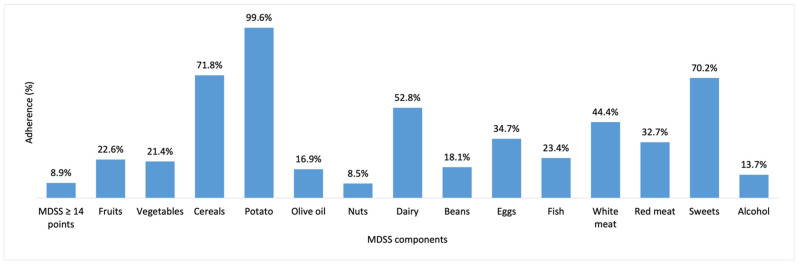
Overall adherence to the MeDi and its components among all study participants. Abbreviations: MDSS, Mediterranean Diet Serving Score.

Out of all study participants, only 24 (8.9%) scored 14 or more points on total MDSS score underlining, exceptionally low adherence to the principles of the MeDi. Overall adherence to the MeDi and its components among all study participants are shown in Figure 2. The highest adherence for each MeDi component was found for potatoes (99.6%), cereals (71.8%), and sweets (70.2%), whereas the lowest was for olive oil (16.9%), alcohol (13.7%), and nuts (8.5%).

**Figure 3:**
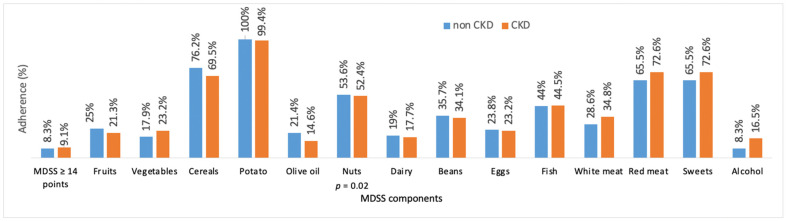
Adherence to the MeDi and its components regarding the presence of CKD. Abbreviations: CKD, participants with chronic kidney disease; non CKD, participants without chronic kidney disease; MDSS, Mediterranean Diet Serving Score *p*-values were obtained with chi-square test (*p* < 0.05).

Among those DDHP with CKD, only 15 (9.1%) scored 14 or more points on the total MDSS score, underlining exceptionally low adherence to the principles of the MeDi. There was no statistically significant difference in overall adherence to the MeDi and its components between DDHP according to CKD presence, with the exception of nut intake (*p* = 0.02) as shown in Figure 3. 

When adjusted for age, gender, BMI, and eGFR statistically significant associations between blood pressure parameters, body composition, and anthropometric parameters with the risk of development of CKD among DDHP were not found as shown in Table 4. A significant association was found between creatinine, phosphorus, proteinuria, and albuminuria and the risk of CKD development as shown in Table 4. Therefore, when adjusted for age, gender, BMI, eGFR, and MDSS, the same significant association was found among DDHP (Table 4).

When adjusted for age, gender, BMI, and eGFR, statistically significant association between food intake adherence according to MeDi and MDSS score with risk of development of CKD among DDHP were not found as shown in Table 5.

## 4. Discussion

To our knowledge, this is the first study to evaluate differences in nutritional status and adherence to MeDi in DDHP regarding the presence of CKD from the Mediterranean region, Dalmatia, Croatia. 

According to the latest evidence, the most important positive effects of the Mediterranean diet on human health include lipid-lowering effect; protection against oxidative stress, inflammation, and platelet aggregation; modification of hormones and growth factors involved in the pathogenesis of cancer; inhibition of nutrient sensing pathways by specific amino acid restriction; and gut microbiota-mediated production of metabolites influencing metabolic health [23]. Considering that AH and DM are major risk factors for CKD occurrence, and the MeDi was found to have a positive impact on the prevention and treatment of both diseases mentioned [24], the focus of our study was to analyze MeDi adherence among DDHP and compare the aforementioned adherence between these specific participants regarding the presence of CKD. 

Out of 248 participants with T2DM and AH included in this study, 164 (66.1%) of them were diagnosed with CKD, with higher disease prevalence in older and male participants. A possible explanation for this finding could be that male sex is associated with an increased risk of CKD, especially in older men [25,26].

Our results indicate that 88.3% of the study population were overweight or obese, which is higher than the European health interview survey (EHIS, 2019) conducted with adults in our country, whose results indicate that 58.5% of women and 73.2% of men were overweight or obese [27]. Prevalence of overweight and obesity among those participants with CKD amounted to a high 87.8%. Although most of the study participants had BMI higher than 25, participants with present CKD had a significantly lower BMI than participants without CKD. Moreover, a significantly lower proportion of adipose tissue and higher muscle mass was noticed in participants with CKD. A possible explanation for these results is that there was a significantly higher proportion of males among those with CKD. Although obesity is not susceptible to gender differences, body composition differs between men and women, indicating that men have more muscle mass, and women have more fat mass. Moreover, men are more likely to accumulate excess adipose tissue in the abdomen, whereas women are more likely to accumulate it in the hips and thighs [28].

Dietary patterns for people with CKD are among the most restrictive diets of all chronic diseases [29]. Therefore, due to limited overlap in foods rich in protein and minerals, such as sodium, potassium, and phosphorus, those patterns are difficult to follow and can contribute to a reduction in overall food intake. Moreover, these restrictions, which are often overwhelming and challenging for patients, can lead to reduced intake of foods that are traditionally considered as healthy, such as fruits, vegetables, whole grains, legumes, and nuts [30]. In the absence of adequate and individual dietary instructions, patients often self-eliminate foods from the diet that can further lead to reduced energy intake and explain differences in the body composition between these two groups of patients [30]. On the other hand, differences might be because participants with CKD are more aware of their kidney disease as part of diabetes and the risk of protein-energy wasting (PEW). Given the significant risk of PEW associated with disease progression, increased BMI in this population is considered as a positive factor [31]. The obesity paradox is thought to play a protective role in patients with CKD regardless of sex, age, and the severity of obesity [31]. 

In addition, a significantly higher value of MCV was found among those participants with CKD. A possible explanation for this finding could be the fact that patients with CKD often have a deficiency of folic acid and vitamin B 12 [32] due to numerous dietary restrictions, such as lower intake of red meat and green leafy vegetables [33,34]. Furthermore, statistically significant lower values of erythrocyte count and hemoglobin levels were found in DDHP with present CKD. Having in mind that nearly all patients with CKD have anemia, erythropoietin deficiency is the predominant cause of anemia in CKD because it is synthesized in kidneys and is responsible for erythropoiesis stimulation [35].

Moreover, statistically significant higher values of urea and creatinine as well as lower value of eGFR were found in DDHP with present CKD. It is well known that renal function deterioration results in elevations of urea and serum creatinine levels and lowering of eGFR [36]. Other biomarkers of renal function are levels of albuminuria and proteinuria in a 24-h urine sample, and statistically higher values were in participants with CKD. Latest KDIGO guidelines use eGFR and albuminuria levels to determine the staging of CKD. Albuminuria is an early and sensitive marker of diabetic nephropathy [37,38], and according to the latest diabetes guidelines, albuminuria screening is recommended on a yearly basis for every patient with T2DM [39]. Statistically significant higher values of potassium and phosphorus were found in DDHP with present CKD. Although potassium and phosphorus levels are well balanced by homeostatic mechanisms, in later stages of chronic kidney disease, hyperkalemia and hyperphosphatemia are frequently present and as such present a great challenges in optimal adjustment of nutritional therapy [40]. 

Furthermore, statistically significant higher values of uric acid, which is another biomarker primarily associated with kidney function, were found in DDHP with present CKD. Elevated levels of uric acid can lead to multiple organ dysfunctions through mechanisms of endothelial dysfunction, vascular smooth muscle cell proliferation, increased IL-6 synthesis, and as well as impairment of nitric oxide production [41].

Among all DDHP, only 52.8% had received some kind of dietary recommendations from healthcare workers prior to nutritional counseling, whereas 49.6% of participants with CKD had received the same recommendations. Moreover, only 19% of non-CKD participants and 12.8% of CKD participants were adherent to the given recommendations. These results are a consequence of the non-existent individual patient-oriented approach and adequate structured nutritional care in our region. Appropriate patient education is of utmost importance not only to accomplish successful nutritional management but also to achieve and maintain patient compliance [13]. Our results showed that family members significantly more often prepare meals for those participants with CKD, which may be due to numerous dietary restrictions, high prevalence of depression [42] and cognitive dysfunction [43], and other associated diseases in this population of patients. Additionally, it is important to note that most of the participants with CKD were elderly and male, and this is the region where women traditionally prepare meals.

Adherence to the MeDi among all study participants was an exceptionally low (8.9%), with an MDSS score of 8.32 ± 3.45, which is consistent with the results obtained on a healthy population from Kolčić et al. [44], underlying poor adherence to the MeDi in southern Dalmatia. Furthermore, similar results were determined in our recent study conducted on kidney transplant recipients from the Dalmatia region, indicating poor adherence to the MeDi principles in this specific population [18].

Several studies have revealed an inverse association between adherence to the MeDi and risk of obesity, cardiovascular diseases, T2DM, as well as all-cause mortality [45,46,47,48,49,50]. Since traditional MeDi contains high amounts of monosaturated fats and omega-3 fatty acids, dietary fibers, polyphenols, vitamins, and antioxidants, the synergic effect of mentioned nutrients with significant impact on WC, HDL, triglycerides, fasting blood glucose, blood pressure, as well as systemic inflammation was noticed [51].

Although 66% of the total study population had CKD, only 9% of them were adherent to the MeDi. There was no difference in the total MeDi adherence between participants with and without CKD. Similar results were noticed in an Australian study conducted on 451 participants with diagnosed CKD [52]. 

Observing each MDSS component separately based on the presence of the CKD, a statistically significant difference was only determined for nut intake. It has been noticed that CKD patients often avoid nut consumption because of their high potassium content, which in turn can lead to hyperkalemia [53]. Serum potassium levels can further be lowered by proper food preparation and using potassium binders that lower the risk of food-induced hyperkalemia [54,55].

Therefore, we did not find associations between adherence to each MeDi component as well as MDSS score with the development of CKD among all study subjects. This could be due to a relatively small number of study participants as well a small number of participants that were adherent to MeDi. 

In contrast to our study, a recent German study found an association between the MeDi and better renal function in CKD participants [56]. In addition, according to the recent systematic review, adherence to the principles of the MeDi was associated with a lower possibility of developing CKD as well as a lower mortality rate among patients with already diagnosed CKD [25]. Additionally, participants with higher compliance to the MeDi principles could have about 50% lower risk of CKD development as shown in a six-year follow-up study [57]. Moreover, as demonstrated in a 15-year observational study, the risk of a rapid decline of renal function was inversely associated with adherence to the MeDi principles [58]. 

According to all these results, there is an urgent need to improve nutritional care in our region, with a special focus on the MeDi for this particularly vulnerable population of the patients.

This study has a few limitations. It was conducted in a single center of tertiary care where a relatively high number of participants from wider geographical regions gravitates. A relatively small number of participants was included, but the sample was representative because it included an especially vulnerable population suffering from both T2DM and AH. Another limitation comes from the cross-sectional design of the study, which disables us from causal conclusions. Furthermore, a very low percentage of participants were adherent to the MeDi, so further analysis was limited. Possible bias in determining MeDi adherence could be caused by self-administration of the questionnaire and unreliable answers regarding intake recall and overestimation or underestimation of food intake although a qualified dietitian was available for any confusing questions. Lastly, data about participants′ income and level of education are lacking.

## 5. Conclusions

Our study showed that a surprisingly small number of patients diagnosed with both T2DM and AH had received any kind of dietary recommendations. Moreover, poor adherence to the MeDi principles was determined in this a population of patients without differences regarding the presence of CKD. It is important to note that this is population where nutrition plays a major role in the treatment of their chronic diseases. Considering all the benefits provided by the MeDi, the results obtained in this study were devastating and indicate further the necessity of structured nutritional care as well as timely education of patients about their dietary needs in a regard to the present diseases. Better nutritional care with special focus on the MeDi for CKD patients could not only reduce the cost of treatment but also might reduce the risk of comorbidities and increase the life expectancy in this population of patients. Furthermore, it could also be a small step in preserving our planet, which is of paramount importance nowadays. Prospective studies with a higher number of participants should be designed to investigate the impact of the MeDi on chronic diseases.

## Figures and Tables

**Table 1:** Basic characteristics of the study population and differences among DDHP with present CKD and without present CKD.

	Non CKD (*n* = 84)	CKD (*n* = 164)	Total (*n* = 248)	*p*-Value *
Age (years), median (IQR)	62 (55–70)	71 (64–76)	68 (60–74)	<0.001
Sex M/F	41/43	102/62	143/105	0.040 ^†^
Duration of DM treatment (years)	9.5 (2–19)	10 (6–20)	10 (5–20)	0.059
Duration of AH treatment (years)	10 (4–20)	13 (8–20)	10 (6–20)	0.164
Smoking, *n* (%)	10 (13.1)	38 (23.8)	48 (19.4)	0.060 ^†^
pSBP (mmHg)	143 (133–160)	147.5 (134–162)	146 (133–160)	0.457
pDBP (mmHg)	94 (85–102)	90 (80–99)	91 (80–100)	0.027
BMI (kg/m^2^), median (IQR)	32.35 (28.6–36.9)	29.6 (27.3–32.7)	30.45 (27.6–34.7)	<0.001
BMI category, *n* (%)				
BMI < 18.5 kg/m^2^	0	2 (1.2)	2 (0.8)	0.005 ^†^
BMI 18.5–24.9 kg/m^2^	9 (10.7)	18 (11.0)	27 (10.9)	
BMI 25–29.9 kg/m^2^	19 (22.6)	69 (42.1)	88 (35.5)	
BMI 30–34.0 kg/m^2^	22 (26.2)	38 (23.2)	60 (24.2)	
BMI 35–39.9 kg/m^2^	16 (19.0)	25 (15.2)	41 (16.5)	
BMI ≥ 40 kg/m^2^	18 (21.4)	12 (7.3)	30 (12.1)	
Anthropometric parameters				
WHtR	0.65 (0.60–0.72)	0.63 (0.58–0.69)	0.63 (0.58–0.70)	0.047
WHR	0.94 (0.89–1.00)	0.97 (0.92–1.00)	0.96 (0.91–1.02)	0.029
Middle upper arm circumference (cm)	33.8 (30.9–37.0)	31.5 (28.8–34.0)	32 (29.1–35.4)	0.001
Hip circumference (cm)	111.3 (105.9–119.4)	118 (109.6–133.9)	113.5 (107.9–123.0)	0.012
Waist circumference (cm)	113.0 (103.9–122.6)	110.0 (101.4–118.0)	110.3 (102.0–119.9)	0.024
Body composition				
Body fat (%)	33.1 (25.7–39.5)	26.9 (21.8–32.3)	28.4 (22.7–36.2)	<0.001
Body fat (kg)	32.6 (24.0–46.7)	23.9 (18.1–31.3)	27.0 (18.7–35.0)	<0.001
Fat free mass (kg)	64.6 (58.1–77.6)	65.9 (58.7–73.8)	65.5 (58.3–74.7)	0.573
Trunk fat mass (kg)	15.5 (12.0–22.3)	13.2 (8.9–17.0)	14.0 (9.7–18.3)	0.001
Trunk fat mass (%)	30.5 (23.4–35.2)	26.3 (20.3–31.1)	27.8 (21.6–32.9)	0.001
Muscle mass (%)	63.6 (57.3–70.8)	69.5 (63.7–74.4)	68.2 (60.4–73.8)	<0.001
Muscle mass (kg)	61.3 (55.2–73.7)	62.6 (55.7–70.2)	62.2 (55.4–71.0)	0.532
Skeletal muscle mass (%)	34.9 (28.4–39.8)	38.4 (33.8–42.8)	37.6 (31.2–41.9)	<0.001
Skeletal muscle mass (kg)	33.2 (26.3–41.4)	34.4 (28.6–39.6)	34.3 (27.7–39.9)	0.880
SMI (Skeletal muscle index)	9.0 (8.1–10.3)	9.1 (8.1–9.9)	9.0 (8.1–10.1)	0.575
Phase angle (°)	5.7 (5.0–6.3)	5.5 (4.8–6.2)	5.6 (4.9–6.3)	0.174

Abbreviations: Non-CKD, participants without present chronic kidney disease; CKD, participants with present chronic kidney disease; M, male; F, female; BMI, body mass index; DM, diabetes mellitus; AH, arterial hypertension; eGFR, estimated glomerular filtration rate using CKD-EPI (mL/min/1.73 m^2^); WHtR, waist-to-height ratio; WHR, waist-to-hip ratio; pSBP, peripheral systolic blood pressure; pDBP, peripheral diastolic blood pressure; SMI, skeletal muscle index. * *p*-values were obtained with Mann–Whitney U test; ^†^ chi-square test.

**Table 2:** Biochemical parameters of the study population and differences among DDHP with present CKD and without present CKD.

	Non CKD (*n* = 84)	CKD (*n* = 164)	Total (*n* = 248)	*p*-Value *
E	4.9 (4.6–5.1)	4.4 (4.0–4.9)	4.5 (4.2–5.0)	<0.001
Hb (g/L)	140 (131–153)	131 (118–144)	134 (122–146)	<0.001
MCV (fL)	87 (84.1–91.1)	89.2 (86.55–93.05)	88.7 (85.8–92.1)	0.004
Urea (mmol/L)	6.3 (4.9–7.6)	11.8 (8.9–15.68)	9.6 (6.6–13.7)	<0.001
Creatinine (mmol/L)	73.5 (62–88.3)	154 (120–201)	122.0 (80.5–170.0)	<0.001
eGFR (mL/min/1.73 m^2^)	84.0 (71.0–96.6)	35.2 (24. 8–48.1)	47.3 (29.0–74.4)	<0.001
HbA1c (%)	6.9 (6.4–7.7)	6.9 (6.3–7.8)	6.9 (6.3–7.7)	0.546
Glucose (mmol/L)	7.4 (6.6–8.9)	7.6 (6.5–9.4)	7.5 (6.5–9.2)	0.983
Alb (g/L)	41.5 (40.0–45.5)	42 (38–45)	42 (39–45)	0.451
Total cholesterol (mmol/L)	5.0 (4.2–5.9)	4.8 (4.2–5.8)	4.9 (4.2–5.8)	0.407
LDL (mmol/L)	2.8 (2.2–3.7)	2.8 (2.1–3.5)	2.8 (2.1–3.6)	0.841
Tgl (mmol/L)	1.9 (1.3–2.5)	1.9 (1.6–2.8)	2.0 (1.4–2.7)	0.239
K (mmol/L)	4.4 (4.1–4.7)	4.5 (4.2–4.9)	4.5 (4.2–4.8)	0.014
Ca (mmol/L)	2.4 (2.3–2.5)	2.4 (2.25–2.5)	2.4 (2.3–2.5)	0.345
P (mmol/L)	1.1 (0.9–1.1)	1.2 (1.0–1.4)	1.1 (1.0–1.3)	0.007
ACR (mg/g)	1.1 (0.7–2.4)	12.7 (1.9–87.4)	3.4 (0.7–22.7)	<0.001
Proteinuria (mg/24 h)	153 (76–218)	902 (204–2062)	496 (136–1488)	<0.001
Albuminuria (mg/24 h)	32 (8–53)	500 (61–1177)	194 (32–990)	<0.001
Uric acid (mmol/L)	397 (327–439)	422 (370–486)	418 (360–476)	0.006

Abbreviations: Non-CKD, participants without present chronic kidney disease; CKD, participants with present chronic kidney disease; E, erythrocyte count; Hb, hemoglobin (g/L); MCV, mean cellular volume (fL); HbA1c, hemoglobin A1c (%); Alb, serum albumin (g/L); LDL, low-density lipoprotein cholesterol (mmol/L); Tgl, triglycerides (mmol/L); K, potassium (mmol/L); Ca, calcium (mmol/L); P, phosphorus (mmol/L); ACR, albumin to creatinine ratio (mg/g). * *p*-values were obtained with Mann–Whitney U test.

**Table 3:** Lifestyle questionnaire and differences regarding the presence of CKD in all study participants.

	Number (%) of Participants			
	Non CKD (*n* = 84)	CKD (*n* = 164)	Total (*n* = 248)	*p*-Value *
Rapid weight changes	22 (26.2)	31 (18.9)	31 (18.9)	0.185
Last body weight measurement				
Do not remember	18 (21.4)	39 (23.8)	57 (23.0)	0.528
Yesterday	18 (21.4)	34 (20.7)	52 (21.0)	
One week ago	28 (33.3)	40 (24.4)	68 (27.4)	
One month ago	16 (19.0)	37 (22.6)	53 (21.4)	
Before more than one month	4 (4.8)	14 (8.5)	18 (7.3)	
Received any kind of dietary recommendation	73 (49.0)	81 (49.4)	131 (52.8)	0.130
Following dietary recommendations				
No	68 (81.0)	143 (87.2)	211 (85.1)	0.281
Yes	16 (19.0)	21 (12.8)	37 (14.9)	
Regular stool	62 (73.8)	128 (78.0)	190 (76.6)	0.455
Presence of nausea	12 (14.3)	26 (15.9)	38 (15.3)	0.746
Loss of appetite	12 (14.3)	28 (17.1)	40 (16.1)	0.327
Alcohol consumption				
No	75 (89.3)	149 (90.9)	224 (90.3)	0.645
Yes	9 (10.7)	15 (9.1)	24 (9.7)	
Physical activity	48 (57.1)	80 (48.8)	128 (51.6)	0.212
Number of meals				
1–2	9 (10.7)	11 (6.7)	20 (8.1)	0.519
2–4	64 (76.2)	133 (81.1)	197 (79.4)	
>4	11 (13.1)	20 (12)	31 (12.5)	
Meal preparation				
Personally	38 (65.5)	40 (37.0)	78 (47.0)	<0.001
Family member	20 (34.5)	68 (63.0)	88 (53.0)	
Eating breakfast	70 (80.3)	142 (86.6)	212 (85.5)	0.491
Eating snacks	47 (56.0)	93 (56.7)	140 (56.5)	0.910
Adding salt to meals				
No	48 (57.1)	44 (26.8)	156 (62.9)	0.343
Yes	36 (42.9)	56 (34.1)	92 (37.1)	
Prescribed with ONS	11 (13.3)	23 (14.1)	34 (13.8)	0.854

Abbreviations: CKD, chronic kidney disease; ONS, oral nutritional support. * *p*-values were obtained with chi-square test.

**Table 4:** Association of measured parameters and risk of development of CKD in DDHP.

	Adjusted for Age, Gender, BMI, and eGFR				Adjusted for Age, Gender, BMI, eGFR, and MDSS			
	Beta	OR	95% CI	*p*-Value	Beta	OR	95% CI	*p*-Value
pSBP (mmHg)	−0.008	0.99	0.98–1.01	0.30	−0.009	0.30	0.98–1.01	0.30
pDBP (mmHg)	0.02	1.02	0.99–1.05	0.20	0.02	1.02	0.99–1.05	0.20
Biochemical parameters								
E	−0.01	0.99	0.54–1.82	0.97	0.005	1.01	0.54–1.87	0.98
Hb (g/L)	−0.01	0.99	0.97–1.01	0.18	−0.02	0.98	0.97–1.01	0.15
MCV (fL)	−0.02	0.98	0.94–1.03	0.39	−0.02	0.98	0.94–1.03	0.37
Urea (mmol/L)	0.002	1.002	0.99–1.02	0.81	0.001	1.001	0.99–1.02	0.85
Creatinine (mmol/L)	0.02	1.02	1.00–1.03	0.01	0.02	1.02	1.01–1.03	0.009
K (mmol/L)	0.17	1.19	0.63–2.25	0.59	0.18	1.19	0.63–2.26	0.59
P (mmol/L)	2.39	10.9	1.14–103.8	0.03	2.42	11.2	1.13–110.45	0.03
ACR (mg/g)	0.007	1.01	0.99–1.03	0.48	0.006	1.01	0.99–1.03	0.48
Proteinuria (mg/24 h)	0.001	1.001	1.0–1.002	0.03	0.001	1.001	1.0–1.002	0.03
Albuminuria (mg/24 h)	0.001	1.001	1.0–1.002	0.03	0.001	1.001	1.0–1.002	0.02
Uric acid (mmol/L)	0.001	1.001	0.99–1.005	0.66	0.001	1.001	0.99–1.005	0.66
Anthropometric parameters								
WHtR	0.106	1.11	0.02–60.88	0.96	0.002	1.002	0.02–48.52	0.99
WHR	1.39	4.03	0.004–3656.2	0.69	1.42	4.13	0.004–1.15	0.69
Middle upper arm circumference (cm)	0.02	1.02	0.92–1.13	0.71	0.02	1.02	0.92–1.13	0.69
Hip circumference (cm)	0.06	1.06	0.97–1.15	0.18	0.06	1.06	0.98–1.15	0.18
Waist circumference (cm)	−0.01	0.98	0.96–1.02	0.42	−0.01	0.99	0.96–1.02	0.38
Body composition								
Body fat (%)	0.006	1.01	0.93–1.09	0.88	0.006	1.01	0.93–1.09	0.89
Body fat (kg)	−0.01	0.99	0.93–1.04	0.63	−0.01	0.99	0.94–1.04	0.63
Trunk fat mass (kg)	0.03	1.03	0.96–1.10	0.42	0.03	1.03	0.96–1.1	0.44
Trunk fat mass (%)	0.001	1.001	0.95–1.05	0.97	0	1.0	0.95–1.05	0.98
Muscle mass (%)	0	1.0	0.95–1.06	0.99	0.002	1.002	0.95–1.06	0.95
Skeletal muscle mass (%)	0.02	1.02	0.95–1.09	0.62	0.02	1.02	0.95–1.09	0.64

Abbreviations: M, male; F, female; BMI, body mass index; DM, diabetes mellitus; AH, arterial hypertension; eGFR, estimated glomerular filtration rate using CKD-EPI (mL/min/1.73 m^2^); WHtR, waist-to-height ratio; WHR, waist-to-hip ratio; pSBP, peripheral systolic blood pressure; pDBP, peripheral diastolic blood pressure; SMI, skeletal muscle index; E, erythrocyte count; Hb, hemoglobin (g/L); MCV, mean cellular volume (fL); HbA1c, hemoglobin A1c (%); Alb, serum albumin (g/L); LDL, low-density lipoprotein cholesterol (mmol/L); Tgl, triglycerides (mmol/L); K, potassium (mmol/L); Ca, calcium (mmol/L); P, phosphorus (mmol/L); ACR, albumin-to-creatinine ratio (mg/g); MDSS, Mediterranean Diet Serving Score; CI, confidence interval; OR, odds ratio.

**Table 5:** Association of food intake adherence according to MeDi and MDSS score with risk of development of CKD in DDHP.

Adjusted for Age, Gender, BMI, and eGFR				
	Beta	OR	95% CI	*p*-Value
Fruits	−0.09	0.92	0.74–1.14	0.43
Vegetables	−0.03	0.97	0.78–1.21	0.81
Cereals	0.04	1.04	0.71–1.53	0.83
Potato	0.12	1.13	0.85–1.49	0.40
Olive oil	0.07	1.07	0.87–1.33	0.51
Nuts	−0.01	0.99	0.81–1.21	0.91
Dairy	0.24	1.27	0.94–1.72	0.11
Beans	0.17	1.18	0.86–1.63	0.31
Eggs	−0.06	0.94	0.73–1.22	0.65
Fish	0.01	1.01	0.67–1.52	0.96
White meat	−0.03	0.97	0.70–1.34	0.85
Red meat	−0.09	0.91	0.70–1.19	0.49
Sweets	−0.09	0.91	0.70–1.18	0.49
Alcohol	0.06	1.07	0.89–1.27	0.48
MDSS, total score	0.09	1.09	0.93–1.29	0.26
MDSS (<14)				
MDSS ≥ 14	−0.23	0.79	0.27–2.30	0.67

Abbreviations: BMI, body mass index; eGFR, estimated glomerular filtration rate using CKD-EPI (mL/min/1.73 m^2^); MDSS, Mediterranean Diet Serving Score; CI, confidence interval; OR, odds ratio.

## Data Availability

Raw data can be found at corresponding author via e-mail: josiparadic1973@gmail.com.
